# Medical nutrition therapy: use of sourdough lactic acid bacteria as a cell factory for delivering functional biomolecules and food ingredients in gluten free bread

**DOI:** 10.1186/1475-2859-10-S1-S15

**Published:** 2011-08-30

**Authors:** Elke K Arendt, Alice Moroni, Emanuele Zannini

**Affiliations:** 1 School of Food and Nutritional Sciences, University College Cork, Western Road, Cork, Ireland; 2 National Food Biotechnology Centre, University College Cork, Cork, Ireland

## Abstract

Celiac disease (CD) is an immune-mediated disease, triggered in genetically susceptible individuals by ingesting gluten from wheat, rye, barley, and other closely related cereal grains. Currently, the estimated prevalence of CD is around 1 % of the population in the western world and medical nutritional therapy (MNT) is the only accepted treatment for celiac disease. To date, the replacement of gluten in bread presents a significant technological challenge for the cereal scientist due to the low baking performance of gluten free products (GF). The increasing demand by the consumer for high quality gluten-free (GF) bread, clean labels and natural products is rising. Sourdough has been used since ancient times for the production of rye and wheat bread, its universal usage can be attributed to the improved quality, nutritional properties and shelf life of sourdough based breads. Consequently, the exploitation of sourdough for the production of GF breads appears tempting. This review will highlight how sourdough LAB can be an efficient cell factory for delivering functional biomolecules and food ingredients to enhance the quality of gluten free bread.

## Celiac disease

**Figure 1:**
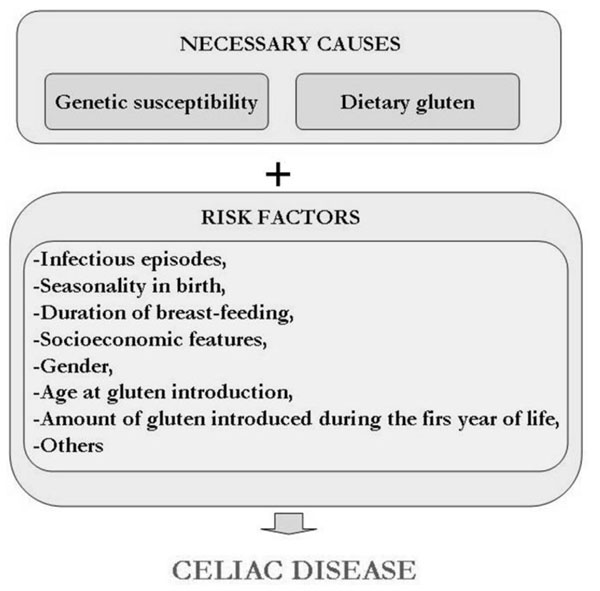
The causes of celiac disease

Celiac disease (CD) is the most common food-induced enteropathy in humans caused by intolerance to wheat gluten and similar proteins originating from barley and rye in genetically susceptible individuals [1]. Previously regarded as a rare disorder, it is now accepted that CD is a major health problem affecting around 1 % of the population in the western world [2,3]. CD has manifested itself in the form of a broad spectrum of clinical symptoms [4-7] (Table 1), which are associated with a large variety of changes in the mucosa of the small intestine [8,9]. The celiac enteropathy is an end-stage lesion that depends on both genetic and environmental factors for expression [6] (Figure 1). To the present day, medical nutrition therapy (MNT) with supportive nutritional care (particularly in relation to iron, calcium and vitamin deficiencies) [10,11] is the only accepted treatment for CD. The current treatment is therefore a strict gluten-free (GF) diet for life.

**Table 1:** Clinical manifestations and related signs of celiac disease^1^

Gastrointestinal Symptoms	Extraintestinal symptoms
Chronic diarrhoea	Infertility or fetal loss
Recurrent pancreaitis	Anaemia
Abdominal distension	Loss of appetite
Abdominal pain	Short stature
Duodenal obstruction	Osteomalacia/osteoporosis
Vomiting	Fatigue
Constipation	Dementia
Flautolence	Weakness (myopothy, neuropthy)
Muscle wasting	Vitamin deficiency
Steatorrhea	Type 1 diabetes
Weight loss	Hypo/hyperthyroidism
Anorexia	Alopecia areata
Bulky, sticky and pale stools	Depression
Failure to thrive	Behavioral changes
	Late-onset puberty
	Epilepsy
	Dermatitis herpetiformis
	Arthritis
	Aphthous stomatis
	Dental enamel hypoplasia
	Cerebellar ataxia
	Myelopathy
	Esophageal reflux

^1^Source : Feighery (1999); Murray (1999); Fasano and Catassi (2001); (Tack et al., 2010).

Gluten is a heterogeneous mixture of wheat storage proteins consisting of gliadins and glutenins. Similar proteins are also present in barley (hordeins) and rye (secalins) and their effect on the health of celiac patient are very well documented. Based on their size, electrophoresis mobility and differential N-terminal sequence, gluten is divided into gliadines which are classified as α, β, γ, and ω –gliadins in addition to the high and low –molecular weight glutenins [12]. Gliadins, also called prolamins due to their high content of the amino acids proline and glutamine, are described as the main triggering factor in CD (Table 2) [13]. Dietary gluten represents a common component of the human diet, not only in wheat bread but also in a wide range of other foods. Due to of its visco-elastic properties, gluten plays a key role in determining the unique baking quality of wheat by being responsible for the water absorption capacity, cohesiveness, viscosity, and elasticity of dough [14].

**Table 2:** Immunogenic gliadin peptides

Amino acid sequences	Position	Immunogenicity
VRVPVPQLQPQNPSQQQPQ	α-gliadin: 1–19	+
QNPSQQQPQEQVPLVQQQ	α-gliadin: 11–28	+
QVPLVQQQQFPGQQQPFPPQ	α -gliadin: 21–40	+
PGQQQPFPPQQPYPQPQPF	α -gliadin: 31–49	+
FPGQQQPFPPQQPYPQPQPF	α -gliadin: 30–49	+
QPYPQPQPFPSQQPYLQL	α -gliadin: 41–58	+
PQPFPSQQPYLQLQPFPQ	α -gliadin: 46–63	+
PQPQLPYPQPQLPY	α -gliadin: 62–75/(a)	+/+++
QLQPFPQPQLPY	α -gliadin: 57–68 (a)	+/+++
QLQPFPQ	α -gliadin: 57–63 (a)	+++
LQLQPFPQPQLPYPQPQLPYPQPQLPYPQPQPF	α -gliadin: 57–89/(a)	+/+++
QLQPFPQPQLPY	α -gliadin: 58–69/(a)	+/+++
PQPQLPYPQPQLPY	α -gliadin: 63–76/(a)	+/+++
PFRPQQPYPQPQPQ	α -gliadin: 93–106 (a)	+
LIFCMDVVLQ	α -gliadin: 123–132	+
QQPLQQYPLGQGSFRPSQQNPQAQG	α -gliadin: 198–222	+
QYPLGQGSFRPSQQNPQA	α -gliadin: 203–220/(a)	+/+
PSGQGSFQPS	α -gliadin: 205–214	-
PSGQGSFQPSQQ	α -gliadin: 205–216/(a)	+/+++
SGQGSFQPSQQN	α –gliadin: 206–217/(a)	+/+++
QGSFQPSQQN	α -gliadin: 208–217/(a)	-/+++
LQPQQPFPQQPQQPYPQQPQ	γ-gliadin: 60–79	+
FPQQPQQPYPQQPQ	γ -gliadin: 66–78	+
FSQPQQQFPQPQ	γ -gliadin: 102–113/(a)	-/+
OQPQQSFPEQQ	γ -gliadin: 134–153/(a)	+/+++
VQGQGIIQPQQPAQL	γ -gliadin: 222–236/(a)	+/+
QQQQPPFSQQQQSPFSQQQQ	glutenin: 40–59/(a)	-/+
QQPPFSQQQQPLPQ	glutenin: 46–60/(a)	-/+
SGQGQRPGQWLQPGQGQQGYYPTSPQQSGQGQQLGQ	glutenin: 707–742/(a)	+/+
PGQGQQGYYPTSPQQSGQ	glutenin: 719–736	+
GYYPTSPQQSGQGQQLGQ	glutenin: 725–742	+
GYYPTSPQQSG	glutenin: 725–735	+
QGYYPTSPQQS	glutenin: 724–734/(a)	-+
QQGYYPTSPQQSG	glutenin: 723–735	+
GQQGYYPTSPQQSG	glutenin: 722–735	+
GQQGYYPTSPQQS	glutenin: 722–734	+

(a): deamidated

Source (Ciccocioppo et al., 2005)

## Gluten-free diet and bread

Total life-long avoidance of gluten ingestion remains the cornerstone treatment for CD. Compliance with a strict gluten-free diet is not easy, because (i) harmful gluten may contaminate food during processing steps, (ii) it is socially limiting, (iii) gluten-free products are generally not widely available and more expensive, are less palatable than conventional wheat bread [15] and (iv) may lead to nutritional deficiencies. Moreover, a marketing review found that most of the gluten-free products were of low quality, exhibiting poor mouth-feel and very often having off-flavours [16]. For these reasons, the replacement of the gluten in gluten-free cereal products is a challenging task for the cereal technologist and bakers.

In the past decades, there has been a significant increasing interest in research on the development of gluten-free bakery products, involving various approaches. These include the use of: (i) gluten-free flours such as, rice, sorghum, oats, buckwheat, amaranth, quinoa, teff, corn (Table 3), (ii) starches, (iii) dairy products [17], (iv) protein supplementation i.e. egg proteins [18], (v) gums and hydrocolloids [19] (vi), dietary fibres [17,20], (vii) the use of functional ingredients, and (vii) alternative technologies such as, enzymatic processing [21,22] and high hydrostatic pressure processing [23]. All these strategies have been showed to improve, to different extents, gluten free cereal products with a final product quality similar to their gluten-containing counterparts. Despite the appealing results obtained so far, the inclusion of these ingredients/additives in bread formulations to improve the quality of GF bread presents several disadvantages. First of all, the GF product prices are excessive and most of the ingredients/additives used represent high-cost components with the average price of GF bread on the market being significantly higher than that of conventional bread (http://www.glutenfree.com/baked-goods/breads/category357) [24]. Additionally, some of these ingredients, e.g. dairy powders, and in particular those with the high lactose-content, are not suitable for coeliacs since a large number of celiac patients are also lactose intolerant, particularly at the early stages of the disease. Furthermore, some additives do not meet the consumers' requirements for natural products. Thus, there is an impelling call for researchers to find alternative technologies for the production of good quality GF bread. In the next paragraphs we address how sourdough lactic acid bacteria can act as “cell factories” for the production of functional biomolecules and food ingredients able to counteract the problems associated with the production of high quality GF breads.

**Table 3:** Grain storage proteins

Storage protein not allowed for CD patient	Storage protein allowed CD patient
Wheat (*Triticum* spp. Including spelt, emmer, farro, einkorn, kamut, dickel, durums)	Amaranth^a^
Rye (*Secale*)	Buckwheat^a^
Triticale (a *Triticum x Secale* cross)	Corn
Barley (*Hordein*)	Millet
Oat *	Quinoa^a^
	Rice
	Sorghum^a^
	Soy^a^
	Legumes^a^
	Teff

^a^These sources contain higher levels of fiber, protein, calcium and iron.

* Most oats have been grown, stored, transported or processed with the gluten-containing grains and are therefore contaminated with gluten.

## GF sourdough fermenting microorganisms

Sourdough is a mixture of flour and water which is fermented with lactic acid bacteria (LAB) and yeasts; these microorganisms determine its characteristics in terms of acid production, aroma and leavening properties [25].

Ecological studies on GF sourdoughs indicate that GF materials harbour novel and competitive LAB and yeasts strains which are not commonly isolated in traditional sourdoughs and could serve as suitable candidates for starter dough development [26-30]. These studies revealed that *Lactobacillus fermentum*, *L. plantarum*, and also *L. paralimentarius* are frequently isolated in GF sourdoughs produced from rice, maize, buckwheat, teff and amaranth. Furthermore, species such as *L. gallinarum*, *L. graminis*, *L sakei* and *Pediococcus pentosaceus*, which are not commonly associated with conventional sourdoughs, were part of the dominant microbiota of the various GF sourdoughs [24]. Since these particular strains are adapted to the various GF-systems, they can be applied as promising cell factory for the delivery of functional biomolecules and food ingredients in gluten free bread.

## Exopolysaccharides (EPS) producing LAB as hydrocolloid replacer in GF system

The addition of hydrocolloids such as xanthan gum, guar gum and hydroxypropylmethylcellulose (HPMC) is essential in gluten-free baking to obtain acceptable product quality in terms of volume, texture, and shelf life [31]. Many lactic acid bacteria (LAB) can produce a wide variety of long-chain sugar polymers called exopolysaccharides (EPS), which are varied in their chemical composition, structure and physical properties [32]. These polysaccharides are synthesised extracellularly from sucrose by glycansucrases, or intracellularly by glycosyltransferases from sugar nucleotide precursors.

LAB isolated from various cereal based sources frequently produce oligo- and homopolysaccharides from sucrose, which can improve the technological as well as the nutritional properties of gluten-free breads acting as prebiotics and hydrocolloids, respectively.

Recently, the applicability of the EPS-producing strains *L. reuteri* LTH5448 and *Weissella cibaria* 10M was investigated in GF sourdoughs [33]. Both strains were shown to be suitable as sourdough fermentation starters for quinoa and sorghum, and during the fermentation were able to produced levan (fructo-oligosaccharides - FOS) and dextran (gluco-oligosaccharides - GOS), respectively. GF breads containing sourdough fermented by *W. cibaria* were softer than the ones containing no EPS. Moreover, GOS produced by *W. cibaria* were not digested by baker’s yeast and they were still present in the final bread. Thus, the consumption of 300 g of sorghum GF bread prepared with *W. cibaria* 10M would allow for a significant intake of prebiotic GOS [33]. Recently, Galle at al. [34] screened EPS-forming *Weissella* strains for their potential use as starter strains in sorghum and wheat sourdoughs. Independent of which strain is used, higher amounts of EPS were formed in sorghum sourdough than in wheat, due to the higher concentration of glucose in the GF flour. In particular, the strains *Weissella kimchii* and *W. cibaria* MG1 produced dextrans in concentrations high enough to be use as potential replacers of non bacteria hydrocolloids, such as guar gum and HPMC in gluten-free sourdoughs bread. All together, these studies indicate that EPS-producing LAB in sourdough could play a promising role for the production of GF products with improved quality characteristics and reduced additives content. However, more research is needed to identify the most suitable EPS for the replacement of hydrocolloids in gluten free cereal products.

## Production of peptidase for gluten detoxification

During endoluminal digestion, gliadins of wheat, rye and barley release a family of peptides rich in Pro and Gln, which are responsible for the inappropriate T cell-mediated immune response associated with celiac disease [35] (Table 2). Recently, sourdough LAB have been considered as cell factories for the production of enzymes able to decrease the toxicity of wheat and rye flours over a long fermentation period (12-24 h). Di Cagno et al. [36] showed that four sourdough LAB strains - *Lactobacillus alimentarius* 15M, *L. brevis* 14G, *L. sanfrancisciensis* 7A and *L. hilgardii* 51B were selected based on their enzyme substrate specificity and ability to hydrolyse the 33-mer peptide (corresponding to a fragment of 57-89 of α 2-gliadin that represents the most potent inducer of gut-derived human T cell lines in patients with celiac disease) [37]. The above mentioned strains where then used for the production of a sourdough containing wheat. Breads were produced by mixing the selected wheat sourdough with untreated GF flours. The final products showed acceptable quality and induced no alterations in the baseline values of celiac patients during in vivo acute challenge test [36]. The same pool of LAB showed also to be effective in reducing the toxicity of rye flour [38] and, when used in association with *L. sanfranciscensis* and fungal protease, produced non-toxic wheat sourdough bread of acceptable quality [39]. Additionally, VSL#3 probiotic preparation (VSL Pharmaceuticals, Gaithesburg, MD), containing *Streptococcus thermophilus*, *Lb. plantarum*, *Lb. acidophilus*, *Lactobacillus casei*, *Lactobacillus delbrueckii spp. bulgaricus*, *Bifidobacterium breve*, *Bifidobacterium longum* and *Bifidobacterium infantis* was also tested successfully by the same authors [40].

However, even if this approach is not directly applicable to the industrial production of gluten-free bread, the results collected so far strongly indicate that selected LAB can be used to degrade any potential contaminant present in gluten-free flours and, at the same time, improve the nutritional properties of GF-breads [41,42].

Beyond the gluten detoxifying activity, sourdough-induced proteolysis was also found to be a key event for delaying staling of GF breads [43,44]. Improved textural properties have been reported for sourdough-based sorghum bread [45]. Nonetheless, more investigations are needed in order to understand which GF flours would be positively influenced by sourdough treatment, which fermentation conditions should be applied and which degree of enzymolysis is required in order to enhance their baking performances.

## Production of antifungal compounds

When conventional wheat bread is compared with GF bread currently on the market, it becomes apparent that the majority of the GF products have a very long shelf life. This increase in shelf life is achieved by using modified atmosphere packaging (MPA) and/or the addition of chemical preservatives [46]. The majority of GF products are based on hydrocolloids, which are essential for structure formation in these products. Hydrocolloids are able to bind a high amount of water which leads to a much higher water activity (aw) in GF breads than in their wheat containing counterparts. This increase in aw leads to a significant reduction in shelf life which can mainly be attributed to mould growth. For this reason the use of MPA and/or chemical preservatives are necessary. The interest in the concept of food biopreservation, which is the control of one organism by another, has increased in recent years. LAB with antifungal activity, preventing growth of bread spoilage fungi, are a promising alternative to chemical preservation [47-50].

To date, only one study has investigated into the use of sourdough to extend the shelf life of GF bread. In this study, Moore et al. [44] used the antifungal strain *L. plantarum* FST 1.7 [48] to produce GF sourdough from a mixture of brown rice, corn starch, buckwheat, and soya flours. Results showed that fermenting 20% of the GF flours with *L. plantarum* FST 1.7 could retard the onset of staling in respect to chemically acidified GF control breads. Furthermore, the sourdough retained its inhibitory activity in the bread, where the growth of *Fusarium culmorum* was retarded by up to 3 days when compared to the control bread. This study clearly indicates that the production of GF sourdough with antifungal properties can be regarded as a valuable alternative to the use of chemical additives for retarding staling and prolonging shelf life of GF breads. However, further research is needed to identify the optimal sourdough starter and fermentation conditions for achieving GF bread of improved shelf life and quality.

## LAB with antimycotoxigenic activity

Different cereals (rice, maize, millet, sorghum) and pseudocereals (amaranth, buckwheat and teff) are widely utilized, with other functional ingredients, in the formulation of gluten-free products, especially for the production of gluten-free bread [45,51-54]. All these alternative grains are mainly produced in tropical and sub tropical regions, where climate and poor storage conditions are conducive to fungal growth and mycotoxin production [55]. Mycotoxin (e. g. Trichothecene, Zearalenone, Fumonisine, Ochratoxin, Aflatoxin, Deoxynivaleon) contamination in maize, rice, sorghum, millet, buckwheat, teff, have been widely reported [53,55-58]. The occurrence of mycotoxins in gluten-free grains is regarded as a major economical problem [59] and is also potentially dangerous particularly for people suffering of CD. LAB, and in particular the species belonging to the genera *Lactobacillus*, have long been known to possess antimycotoxigenic activity against the most harmful mycotoxins like Zearalenone, Fumonisine, Ochratoxin, Aflatoxin and Deoxynivaleon.

El-Nezami et al.[60] report, that two food-grade *Lactobacillus rhamnosus* GG and *L. rhamnosus* LC705 were able to reduce the contamination of zearelenone (ZEN) and its derivates α’-zearalenol (α’-ZOL) by up to 55% (w/w) using binding sites on the bacteria surface. When the two toxins were tested in combinations, binding of individual toxins was compromised indicating the possibility of the two toxins sharing similar surface binding sites. Similarly, *L. rhamnosus strains* GG and LC705 are also shown to effectively bind aflatoxin B1 [60]. Additionally, many other strains of LAB, such as *L. acidophilus* VM20 [61], *L. acidophilus* CH-5, *L. plantarum* BS, *L. brevis* and *L. sanfrancisciensis *[62] have been reported to bind ochratoxin A (OTA) in a strain specific manner causing its decrease by up to 95% . This allows reduction in the absorption of these toxins from the intestine and hence reducing their estrogenic effects in humans.

Thus, even though further studies in gluten-free bread systems are required, the potential antimycotoxigenic activity of LAB places them in a promising position for developing a new approach for detoxification of mycotoxins in GF products.

## Phytase producing LAB

Phytic acid is the major storage form of phosphorous in grains and it binds minerals such as Ca, Fe, K, Mg, Mn and Zn, and therefore making them insoluble and thus unavailable for adsorption in the intestinal tract of humans [63]. Phytic acid is therefore considered an antinutritional factor, especially for celiac patients who suffer from micronutrient deficiencies. GF flours/ingredients show a wide range of phytate contents, examples are: 0.12 % rice, 0.25% pearl millet, 0.47 amaranth, 0.70% teff, 0.77% lupin, 0.92% corn, 1.13% oats, 1.18% quinoa, 1.33% soybean [64]. In wheat grain the level of phytate is around 1.2%. Since phytate is mainly found in the aleurone layer, the content of phytate in a specific flour is very much depends on the milling regime applied. During bread-making, phytic acid can be degraded by phytases whose activity is influenced by temperature, pH, fermentation time and by the presence of certain additives [65]. Studies conducted on whole wheat show that the moderate decrease in pH caused by sourdough fermentation is sufficient to significantly reduce the phytate content of bread produced from whole wheat flour. Recent studies also showed that phytate can be synthesized by microorganisms, e.g LAB [66] and yeast [67].

De Angelis et al., [66] reported a reduction in phytic acid content of about 64-74% in a wheat dough fermented with *L. sanfranciscensis* CB1 compared to a control sourdough. To date, no work has been published on phytase activity in GF sourdough breads. Yet, phytase activity has been investigated during fermentation of some GF crops. In particular, fermentation of sorghum and pearl millet was shown to induce a decrease in the phytic acid content and two phytase-positive strains, i.e., *L. plantarum* and *L. fermentum*, were isolated from fermenting pearl millet [68].

## Generation of aroma compounds using LAB

The addition of sourdough to bread product can strongly influence the flavour profile of the bread. This flavour modification is dependent on the raw material, type of starter cultures, fermentation and baking condition applied [69]. Among these factors, LAB and yeast play a pivotal role in the generation of volatile metabolites in the final cereal products. The fermentation of GF flours by LAB has been shown to induce the production of flavour compounds. One example is the fermentation of sorghum for the production of *towga* where different flavouring compounds were generated during the fermentation. The flavour changes were attributed to the production of alcohol and diacetyl [70]. Diacetyl was produced in high concentration when the fermentation was carried out with *L. plantarum* and *Pediococcus pentosaceus* and alcohols were produced in significant amount if *Issatchenkia orientalis* was used in combination with *L.brevis* or *L.plantarum*. The *L. plantarum* /yeasts co-fermentation also induced the production of aldehydes. Finally, Edema and Sanni [29] showed that maize flours fermented with mixed cultures containing *L. plantarum* lead to an increased diacetyl content when compared to a control. Thus, even if further studies are required, sourdough technology might be a promising tool to produce flavour enhancing compounds which will improve the poor sensorial quality of GF breads.

## Production of bioactive compounds

Sourdough fermentation is well recognized as a useful aid rendering cereal products palatable. Moreover, the sourdough process represents an important tool in increasing the extractability of bioactive compounds from various raw materials or in releasing functional biomolecules which are part of the LAB/yeast metabolism. However, beyond the potential of sourdough fermentation, the type of raw material (cereal, pseudocereals, and legumes) used is seen of key importance for the optimal delivery of bioactive compounds for human nutrition.

To date, only one research study has investigated the use of LAB to ferment GF flours for the production of functional bread enriched with bioactive compounds. In this study Coda et al., [71] used *L. plantarum* C48 and *Lactococcuslactics* subsp. *lactis* PU1, selected for the capacity to synthesizing GABA ( γ- Aminobutyric acid), through sourdough fermentation of common wheat, durum wheat, rye, spelt, oat, buckwheat, rice, amaranth, millet, chickpea, soy and quinoa flours. γ- Aminobutyric acid (GABA), acts as the chief inhibitory neurotransmitter of the central nervous system [72]. Other physiological functions of GABA are induction of anti-hypertensive, prevention of diabetes, diuretic and tranquiliser effects [73]. The highest biosynthesis of GABA was detected when buckwheat (643 ± 13 mg/Kg) and quinoa (415 ± 10 mg/Kg) were fermented with *Lb. plantarum* C48. *Lc. lactis* subsp. *lactis* PU1 revealed the best results when amaranth (816 ± 11 mg/Kg) and chickpea (1031 ± 9 mg/Kg) where used as substrates. A blend of selected flours was also fermented with two GABA-producing strains; the best performance was found when *Lb. plantarum* C48 was applied with a GABA production equal to 989 ± 10 mg/kg. On the contrary, when common wheat and durum wheat flours were used as a substrate, *Lc. lactis* subsp. *lactis* PU1 showed the worse performance producing 70 ± 15 mg/Kg and 84 ± 26 mg /Kg of GABA respectively.

In conclusion, the use of a blend of buckwheat, amaranth, chickpea and quinoa flours subjected to sourdough fermentation by selected GABA-producing strains represent a promising potential tool for enhancing the nutritional quality of GF-bread.

## Conclusions

Many factors have contributed to the increased prevalence of celiac disease, which has emerged as the most common food intolerance worldwide that can be diagnosed at all ages. Even though, in the past decade, an impressive effort has been made to development potential therapeutic solutions for CD [74], the only currently available and safe treatment for CD consists of the dietary exclusion of grains containing gluten and the supportive nutritional care in case of mineral and vitamins deficiencies in celiac patients [10]. Gluten is an essential structure-building protein, contributing to the appearance, crumb structure, and consumer acceptability of many baked products. Therefore, the biggest challenge for food scientists and bakers in the area of GF products is probably the production of high quality GF bread. Sourdough fermentation positively influences all aspects of bread quality such as texture, aroma, nutritional properties and shelf life. Recently, sourdough has been successfully applied for the improvement of the quality of GF bread due to the complex metabolic activity of the sourdough lactic acid bacteria. The examples presented in this review demonstrate that LAB might be considered as “burgeoning” cell factories for the delivering of functional biomolecules and food ingredients for the production of high quality GF cereal products.

## Competing interests

The authors declare that they have no competing interest.

## Authors' contributions

EKA received the invitation, critically revised and commented the manuscript. AM contributed to the content of the review article. EZ designed and drafted the manuscript.

## References

[B1] Goggins M Kelleher D Celiac disease and other nutrient related injuries to the gastrointestinal tract Am J Gastroenterol 1994 89 S2 17 8048412

[B2] Catassi C Kryszak D Bhatti B Sturgeon C Helzlsouer K Clipp SL Gelfond D Puppa E Sferruzza A Fasano A Natural history of celiac disease autoimmunity in a USA cohort followed since 1974 Ann Med 2010 42 530 8 10.3109/07853890.2010.514285 20868314

[B3] Mustalahti K Catassi C Reunanen A Fabiani E Heier M McMillan S Murray L Metzger MH Gasparin M Bravi E Maki M The prevalence of celiac disease in Europe: Results of a centralized, international mass screening project Ann Med 2010 42 587 595 10.3109/07853890.2010.505931 21070098

[B4] Feighery C Fortnightly review: coeliac disease BMJ 1999 319 236 9 10417090 10.1136/bmj.319.7204.236 PMC1116331

[B5] Murray JA The widening spectrum of celiac disease Am J Clin Nutr 1999 69 354 65 10075317 10.1093/ajcn/69.3.354

[B6] Fasano A Catassi C Current approaches to diagnosis and treatment of celiac disease: an evolving spectrum Gastroenterology 2001 120 636 51 10.1053/gast.2001.22123 11179241

[B7] Farrell RJ Kelly CP Celiac sprue N Engl J Med 2002 346 180 8 10.1056/NEJMra010852 11796853

[B8] Kaukinen K Maki M Partanen J Sievanen H Collin P Celiac disease without villous atrophy: revision of criteria called for Dig Dis Sci 2001 46 879 87 10.1023/A:1010729207320 11330428

[B9] WAHAB PJ CRUSIUS JB MEIJER JW MULDER CJ Gluten challenge in borderline gluten-sensitive enteropathy Am J Gastroenterol 2001 96 1464 9 10.1111/j.1572-0241.2001.03812.x 11374684

[B10] Hopman EG Le Cessie S Von Blomberg BM Mearin ML Nutritional management of the gluten-free diet in young people with celiac disease in The Netherlands J Pediatr Gastroenterol Nutr 2006 43 102 8 10.1097/01.mpg.0000228102.89454.eb 16819385

[B11] Tack GJ Verbeek WHM Schreurs MWJ Mulder CJJ The spectrum of celiac disease: epidemiology, clinical aspects and treatment Nature Reviews Gastroenterology & Hepatology 2010 7 204 213 10.1038/nrgastro.2010.23 20212505

[B12] Koning F Gilissen L Wijmenga C Gluten: a two-edged sword. Immunopathogenesis of celiac disease Springer Semin Immunopathol 2005 27 217 32 10.1007/s00281-005-0203-9 16091925

[B13] Ciccocioppo R Di Sabatino A Corazza GR The immune recognition of gluten in coeliac disease Clinical & Experimental Immunology 2005 140 408 416 10.1111/j.1365-2249.2005.02783.x 15932501 PMC1809391

[B14] WIESER H Chemistry of gluten proteins Food Microbiol 2007 24 115 9 10.1016/j.fm.2006.07.004 17008153

[B15] Arendt EK Morrissey A Moore MM Dal Bello F Elke, K. A, Fabio Dal, B. Application of dairy ingredients in gluten-free food Gluten-Free Cereal Products and Beverages 2008 San Diego: Academic Press

[B16] Arendt EK O’ Brien CM Schober TJ Gallagher E Gormley TR Development of gluten-free cereal products Farm & Food 2002 21 2721779889

[B17] Gallagher E Gormley TR Arendt EK Recent advances in the formulation of gluten-free cereal-based products Trends in Food Science & Technology 2004 15 143 152 10.1016/j.tifs.2003.09.01221773735

[B18] Ibanoglu E Ercelebi EA Thermal denaturation and functional properties of egg proteins in the presence of hydrocolloid gums Food Chemistry 2007 101 626 633 10.1016/j.foodchem.2006.01.056

[B19] Schober TJ Messerschmidt M Bean SR Park SH Arendt EK Gluten-free bread from sorghum: Quality differences among hybrids Cereal Chemistry 2005 82 394 404 10.1094/CC-82-0394

[B20] Hager AS Ryan LAM Schwab C Ganzle MG O'Doherty JV Arendt EK Influence of the soluble fibres inulin and oat beta-glucan on quality of dough and bread European Food Research and Technology 2011 232 405 413 10.1007/s00217-010-1409-1

[B21] Renzetti S Courtin CM Delcour JA Arendt EK Oxidative and proteolytic enzyme preparations as promising improvers for oat bread formulations: Rheological, biochemical and microstructural background Food Chemistry 2010 119 1465 1473 10.1016/j.foodchem.2009.09.028

[B22] Renzetti S Behr J Vogel RF Arendt EK Transglutaminase polymerisation of buckwheat (Fagopyrum esculentum Moench) proteins Journal of Cereal Science 2008 48 747 754 10.1016/j.jcs.2008.04.005

[B23] Vallons KJR Ryan LAM Arendt EK Promoting structure formation by high pressure in gluten-free flours LWT - Food Science and Technology 2010 in press Corrected Proof

[B24] Moroni AV Dal Bello F Arendt EK Sourdough in gluten-free bread-making: An ancient technology to solve a novel issue? Food Microbiology 2009 26 676 684 10.1016/j.fm.2009.07.001 19747600

[B25] Hammes WP Gänzle MG Woods, B. J. B. Sourdough breads and related products Microbiology of Fermented Foods 1998 London Blackie Academic/Professional

[B26] Moroni AV Arendt EK Morrissey JP Dal Bello F Development of buckwheat and teff sourdoughs with the use of commercial starters International Journal of Food Microbiology 2010 142 142 148 10.1016/j.ijfoodmicro.2010.06.014 20643489

[B27] Vogelmann SA Seitter M Singer U Brandt MJ Hertel C Adaptability of lactic acid bacteria and yeasts to sourdoughs prepared from cereals, pseudocereals and cassava and use of competitive strains as starters International Journal of Food Microbiology 2009 130 205 212 10.1016/j.ijfoodmicro.2009.01.020 19239979

[B28] Sterr Y Weiss A Schmidt H Evaluation of lactic acid bacteria for sourdough fermentation of amaranth International Journal of Food Microbiology 2009 136 75 82 10.1016/j.ijfoodmicro.2009.09.006 19783060

[B29] EDEMA MO SANNI AI Functional properties of selected starter cultures for sour maize bread Food Microbiology 2008 25 616 625 10.1016/j.fm.2007.12.006 18456117

[B30] Meroth CB Hammes WP Hertel C Characterisation of the microbiota of rice sourdoughs and description of Lactobacilius spicheri sp nov Systematic and Applied Microbiology 2004 27 151 159 10.1078/072320204322881763 15046303

[B31] Lazaridou A Duta D Papageorgiou M Belc N Biliaderis CG Effects of hydrocolloids on dough rheology and bread quality parameters in gluten-free formulations Journal of Food Engineering 2007 79 1033 1047 10.1016/j.jfoodeng.2006.03.032

[B32] De Vuyst L Degeest B Heteropolysaccharides from lactic acid bacteria Fems Microbiology Reviews 1999 23 153 177 10234843 10.1111/j.1574-6976.1999.tb00395.x

[B33] Schwab C Mastrangelo M Corsetti A Ganzle M Formation of oligosaccharides and polysaccharides by Lactobacillus reuteri LTH5448 and Weissella cibaria 10M in sorghum sourdoughs Cereal Chemistry 2008 85 679 684 10.1094/CCHEM-85-5-0679

[B34] Galle S Schwab C Arendt E Ganzle M Exopolysaccharide-Forming Weissella Strains as Starter Cultures for Sorghum and Wheat Sourdoughs J Agr Food Chem 2010 58 9 5834 5841 10.1021/jf1002683 20405917

[B35] Sollid LM Khosla C Future therapeutic options for celiac disease Nature Clinical Practice Gastroenterology & Hepatology 2005 2 140 147 10.1038/ncpgasthep0111 16265155

[B36] Di Cagno R De Angelis M Auricchio S Greco L Clarke C De Vincenzi M Giovanni C D'Archivio M Landolfo F Parrilli G Minervini F Arendt E Gobbetti M Sourdough Bead Made from Wheat and Nontoxic Flours and Started with Selected Lactobacilli Is Tolerated in Celiac Sprue Patients Applied and Environmental Microbiology 2004 70 1088 1096 10.1128/AEM.70.2.1088-1096.2004 14766592 PMC348803

[B37] Shan L Molberg O Parrot I Hausch F Filiz F Gray GM Sollid LM Khosla C Structural basis for gluten intolerance in Celiac sprue Science 2002 297 2275 2279 10.1126/science.1074129 12351792

[B38] De Angelis M Coda R Silano M Minervini F Rizzello CG Di Cagno R Vicentini O De Vincenzi M Gobbetti M Fermentation by selected sourdough lactic acid bacteria to decrease coeliac intolerance to rye flour Journal of Cereal Science 2006 43 301 314 10.1016/j.jcs.2005.12.008

[B39] Rizzello CG De Angelis M Di Cagno R Camarca A Silano M Losito A De Vincenzi M De Bari MD Palmisano F Maurano F Gianfrani C Gobbetti M Highly efficient gluten degradation by lactobacilli and fungal proteases during food processing: New perspectives for celiac disease Applied and Environmental Microbiology 2007 73 4499 4507 10.1128/AEM.00260-07 17513580 PMC1932817

[B40] De Angelis M Rizzello CG Fasano A Clemente MG De Simone C Silano M VSL#3 proiotic preparation has the capacity to hydrolize gliadin polypeptides responsible for celiac sprue Biochimica and Biophysica Acta2006b 1762 80 93 10.1016/j.bbadis.2005.09.008 16311022

[B41] Giuliani GM Benedusi A Di Cagno R De Angelis MLA Gobbetti M Miscela di batteri lattici per la preparazione di prodotti da forno senza glutine 2006

[B42] Di Cagno R Rizzello CG De Angelis M Cassone A Giuliani G Benedusi A Limitone A Surico RF Gobbetti M Use of selected sourdough strains of Lactobacillus for removing gluten and enhancing the nutritional properties of gluten-free bread Journal of Food Protection 2008 71 1491 1495 18680953 10.4315/0362-028x-71.7.1491

[B43] Moore MM Juga B Schober TJ Arendt EK Effect of lactic acid bacteria on properties of gluten-free sourdoughs, batters, and quality and ultrastructure of gluten-free bread Cereal Chemistry 2007 84 357 364 10.1094/CCHEM-84-4-0357

[B44] Moore MM Dal Bello F Arendt EK Sourdough fermented by Lactobacillus plantarum FST 1.7 improves the quality and shelf life of gluten-free bread European Food Research and Technology 2008 226 1309 1316 10.1007/s00217-007-0659-z

[B45] Schober TJ Bean SR Boyle DL Gluten-free sorghum bread improved by sourdough fermentation: Biochemical, rheological, and microstructural background Journal of Agricultural and Food Chemistry 2007 55 5137 5146 10.1021/jf0704155 17536829

[B46] Gallagher E Kunkel A Gormley TR Arendt EK The effect of dairy and rice powder addition on loaf and crumb characteristics, and on shelf life (intermediate and long-term) of gluten-free breads stored in a modified atmosphere European Food Research and Technology 2003 218 44 48 10.1007/s00217-003-0818-9

[B47] Ryan LAM Zannini E Dal Bello F Pawlowska A Koehler P Arendt EK Lactobacillus amylovorus DSM 19280 as a novel food-grade antifungal agent for bakery products International Journal of Food Microbiology 2011 146 276 283 10.1016/j.ijfoodmicro.2011.02.036 21429613

[B48] Dal Bello F Clarke CI Ryan LAM Ulmer H Schober TJ Ström K Sjögren J Van Sinderen D Schnürer J Arendt EK Improvement of the quality and shelf life of wheat bread by fermentation with the antifungal strain Lactobacillus plantarum FST 1.7 Journal of Cereal Science 2007 45 309 318 10.1016/j.jcs.2006.09.004

[B49] Lavermicocca P Valerio F Visconti A Antifungal activity of phenyllactic acid against molds isolated from bakery products Applied and Environmental Microbiology 2003 69 634 640 10.1128/AEM.69.1.634-640.2003 12514051 PMC152452

[B50] Gerez CL Torino MI Rollan G Font de Valdez G Prevention of bread mould spoilage by using lactic acid bacteria with antifungal properties Food Control 2009 20 144 148 10.1016/j.foodcont.2008.03.005

[B51] Schoenlechner R Siebenhandl S Berghofer E Arendt EK, D. B. F. Pseudocereals Gluten-free cereal products and beverages 2008 London: Academic Press

[B52] Rossel CM Marco C Arendt EK, D. B. F. Rice Gluten-free cereal products and beverages 2008 London: Academic Press

[B53] Tanaka K Sago Y Zheng Y Nakagawa H Kushiro M Mycotoxins in rice International Journal of Food Microbiology 2007 119 59 66 10.1016/j.ijfoodmicro.2007.08.002 17913273

[B54] Schoenlechner R Siebenhandl S Berghofer E Elke, K. A, Fabio Dal, B. Pseudocereals Gluten-Free Cereal Products and Beverages 2008 San Diego: Academic Press

[B55] Reddy KRN Abbas HK Abel CA Shier WT Oliveira CAF Raghavender CR Mycotoxin contamination of commercially important agricultural commodities Toxin Reviews 2009 28 154 168 10.1080/15569540903092050

[B56] Ayalew A Fehrmann H Lepschy J Beck R Abate D Natural occurrence of mycotoxins in staple cereals from Ethiopia Mycopathologia 2006 162 57 63 10.1007/s11046-006-0027-8 16830193

[B57] Schober TJ Bean SR ARENDT EK, D. B. F. Sorghum and maize Gluten-free cereal products and beverages2008a London: Academic Press 550

[B58] Taylor JRN Emmambux MN ELKE, K. A, FABIO DAL, B. Gluten-free foods and beverages from millets Gluten-Free Cereal Products and Beverages 2008 San Diego: Academic Press

[B59] Dalie DKD Deschamps AM Richard-Forget F Lactic acid bacteria - Potential for control of mould growth and mycotoxins: A review Food Control 2010 21 370 380 10.1016/j.foodcont.2009.07.011

[B60] El-Nezami H Polychronaki N Salminen S Mykkanen H Binding rather than metabolism may explain the interaction of two food-grade Lactobacillus strains with zearalenone and its derivative alpha-zearalenol Applied and Environmental Microbiology 2002 68 3545 3549 10.1128/AEM.68.7.3545-3549.2002 12089040 PMC126820

[B61] Fuchs S Sontag G Stidl R Ehrlich V Kundi M Knasmüller S Detoxification of patulin and ochratoxin A, two abundant mycotoxins, by lactic acid bacteria Food and Chemical Toxicology 2008 46 1398 1407 10.1016/j.fct.2007.10.008 18061329

[B62] Piotrowska M Zakowska Z The elimination of ochratoxin A by lactic acid bacteria strains Pol J Microbiol 2005 54 279 86 16599298

[B63] Bohn L Meyer AS Rasmussen SK Phytate: Impact on environment and human nutrition. A challenge for molecular breeding Journal of Zhejiang University: Science B 2008 9 165 191 18357620 10.1631/jzus.B0710640 PMC2266880

[B64] Wrigley C Corke H Walker C Encyclopedia of Grain Science, 2004 Oxford, UK, Academic Press

[B65] Lopez HW Krespine V Guy G Messager A Demigne C Remesy C Prolonged fermentation of whole wheat sourdough reduces phytate level and increases soluble magnesium Journal of Agricultural and Food Chemistry 2001 49 2657 2662 10.1021/jf001255z 11368651

[B66] De Angelis M Gallo G Corbo MR McSweeney PLH Faccia M Giovine M Gobbetti M Phytase activity in sourdough lactic acid bacteria: Purification and characterization of a phytase from Lactobacillus sanfranciscensis CB1 International Journal of Food Microbiology 2003 87 259 270 10.1016/S0168-1605(03)00072-2 14527798

[B67] Türk M Sandberg AS Carlsson NG Andlid T Inositol hexaphosphate hydrolysis by Baker's yeast. Capacity, kinetics, and degradation products Journal of Agricultural and Food Chemistry 2000 48 100 104 10.1021/jf9901892 10637059

[B68] Songre-Ouattata LT Mouquet-Rivier C Icard-Verniere C Rochette I Diawara B Guyot JP Potential of amylolytic lactic acid bacteria to replace the use of malt for partial starch hydrolysis to produce African fermented pearl millet gruel fortified with groundnut International Journal of Food Microbiology 2009 130 258 264 10.1016/j.ijfoodmicro.2009.02.002 19246113

[B69] Gobbetti M The sourdough microflora: Interactions of lactic acid bacteria and yeasts Trends in Food Science & Technology 1998 9 267 274 10.1016/S0924-2244(98)00053-321773735

[B70] Mugula JK Nnko SAM Narvhus JA Sørhaug T Microbiological and fermentation characteristics of togwa, a Tanzanian fermented food International Journal of Food Microbiology 2003 80 187 199 10.1016/S0168-1605(02)00141-1 12423921

[B71] Coda R Rizzello CG Gobbetti M Use of sourdough fermentation and pseudo-cereals and leguminous flours for the making of a functional bread enriched of [gamma]-aminobutyric acid (GABA) International Journal of Food Microbiology 2010 137 236 245 10.1016/j.ijfoodmicro.2009.12.010 20071045

[B72] Krnjevic K Chemical Nature of Synaptic Transmission in Vertebrates Physiological Reviews 1974 54 418 540

[B73] Wong CGT Bottiglieri T Snead OC GABA, gamma-hydroxybutyric acid, and neurological disease Annals of Neurology 2003 54 S3 S12 12891648 10.1002/ana.10696

[B74] Lerner A New therapeutic strategies for celiac disease Autoimmun Rev 2010 9 144 7 10.1016/j.autrev.2009.05.002 19427921

